# Analysis of risk factors for stenosis after laparoscopic pyeloplasty in the treatment of ureteropelvic junction obstruction

**DOI:** 10.1007/s11255-023-03906-5

**Published:** 2024-01-20

**Authors:** Ruilong Chen, Chao Jiang, Xiang Li, Chao Yang, Tengfei Zhu, Yi Wang

**Affiliations:** Department of Urology, Anhui Medical University Second Hospital, Hefei Economic and Technological Development Zone, No. 678 Furong Road, Hefei, 230601 Anhui China

**Keywords:** Ureteropelvic junction obstruction, Pyeloplasty, Risk factor analysis

## Abstract

**Background:**

Laparoscopic ureteroplasty is an effective method for managing ureteropelvic junction obstruction. Despite its high success rate, there remains a subset of patients who do not experience improvement in the hydrops.

**Methods:**

The study retrospectively analyzed the data of 143 patients with ureteropelvic junction obstruction (UPJO) who underwent laparoscopic pyeloplasty (LP) in our hospital from January 2015 to May 2022. Logistic regression was used to analyze the risk factors of recurrence stenosis after UPJO.

**Results:**

Out of these patients, 119 had complete clinical data and follow-up records. Among these patients, restenosis occurred in nine cases after the operation. There was a significant statistical difference in blood loss (*P* < 0.05). Univariate and multivariate logistic regression analysis revealed that the preoperative separation degree of the renal pelvis, cystatin C, and intraoperative blood loss were potential risk factors for recurrent stenosis after primary LP. When divided by split renal function (SRF), the odds ratio (OR) was 7.850 (*P* = 0.044), indicating that it was an independent risk factor for postoperative restenosis. Similarly, the OR for stenotic segment length was 0.025 (*P* = 0.011), also indicating it as an independent risk factor for restenosis. The areas under the receiver operating characteristic curve for stenotic segment length and SRF were 0.9056 and 0.7697, respectively.

**Conclusion:**

In our study, we identified that preoperative renal pelvis separation, cystatin C, and intraoperative blood loss were potential risk factors for postoperative restenosis. SRF and stenosis segment length were independent risk factors for postoperative restenosis.

## Introduction

Ureteropelvic junction obstruction (UPJO) is a urinary system disease characterized by the narrowing of the junction between the renal pelvis and the ureter. This condition can lead to urinary tract infections, urinary stones, waist and abdomen pain, and other symptoms, ultimately resulting in poor urination. UPJO can manifest clinical symptoms at any stage of a person’s life, with many patients being diagnosed in adulthood. In addition, UPJO can also develop as a result of trauma, stones, and other factors [1, 2].

Anderson–Hynes dismembered pyeloplasty is widely recognized as the gold standard for treating UPJO, boasting a success rate of approximately 90–100% [3, 4]. Since the first reported case of laparoscopic pyeloplasty (LP) in 1993, an increasing number of scholars have explored the use of minimally invasive surgery for UPJO treatment [5]. Literature reports suggest that minimally invasive surgery yields comparable clinical efficacy to open surgery, albeit with potentially longer operation times [6]. Recent literature indicates that LP has emerged as a reliable treatment option for ureteropelvic junction obstruction [7, 7, 8].

Despite advancements in technology and increased expertise, the success rate of pyeloplasty continues to improve. However, it is observed that approximately 2.5–10% of patients experience relapse after the initial operation [10]. Moreover, it is important to note that the recurrence rate of actual surgery may be higher due to insufficient follow-up time [11]. This study aims to investigate the causes of recurrent stenosis after primary LP for ureteropelvic junction obstruction. By analyzing related risk factors, the findings of this study will contribute to the development of effective clinical treatment strategies.

## Methods

### Study population

This retrospective study analyzed a total of 143 UPJO patients admitted to the Second Affiliated Hospital of Anhui Medical University from January 2015 to May 2022. All patients underwent LP, and 119 cases met the inclusion criteria.

The inclusion criteria were as follows:

1. Preoperative enhanced computed tomography urography, urinary system B-ultrasound, radionuclide renal imaging, and other examinations were performed to confirm the diagnosis of UPJO.
2. Successful completion of LP.
3. Availability of complete data and a follow-up period of at least 6 months. The exclusion criteria were as follows:
4. Patients with a duplicate kidney or a solitary kidney.
5. Patients with bilateral stenosis.
6. Patients with incomplete data or lost to follow-up after the operation.

All the enrolled patients were divided into two groups based on their postoperative outcomes: the postoperative hydronephrosis recovery group and the restenosis group. Postoperative restenosis was defined as the absence of significant relief or even worsening of symptoms such as postoperative low back pain and/or the need for surgical treatment due to recurrent obstruction at the ureteropelvic junction site indicated by postoperative imaging examination.

The collected data included preoperative general information such as gender, age, and body mass index (BMI). Perioperative data included the length of stenosis, length of operation, and complications. Complications such as urinary tract infection, anastomotic leakage, and intestinal obstruction were recorded. Postoperative recovery was assessed by recording the time of indwelling ureteral stent and conducting follow-up examinations (urinary system ultrasound, computed tomography, urography) at 3 months and 6 months after stent removal to monitor changes in hydronephrosis. This study adhered to the principles of the Declaration of Helsinki and was approved by the Ethics Committee of the Second Affiliated Hospital of Anhui Medical University. Since it was a retrospective analysis, individual consent was not required.

### Surgical technique

The surgical procedures were performed by experienced senior chief doctors with over 15 years of medical experience. The surgical operation area was established using the three-hole method, and the ureteropelvic junction was released. The location of the stenosis was determined and the ureter was split longitudinally until it reached the normal ureteral mucosa. The length of the narrowed segment was measured and removed, and the distal ureter was longitudinally split by 1.5–2.0 cm. The dilated hydrops in the proximal end of the renal pelvis was excised, followed by the insertion of a double J-tube. Finally, the two ends were continuously anastomosed.

### Statistical analysis

The data were analyzed using SPSS software (version 26.0) for statistical analysis. Scatter plots and receiver operating characteristic (ROC) curves were drawn using GraphPad Prism software (version 8.0). Categorical variables such as gender, affected side, and history of ureteral surgery were presented as numbers and percentages, and the Chi-square test was used for group comparisons. Measurement data such as BMI, preoperative renal pelvis separation, and length of stenosis were normally distributed and expressed as mean ± standard deviation. Independent sample *t* test was used for comparison between groups. The indwelling time of the double J-tube, the indwelling time of the drainage tube, and the amount of blood loss during the operation were not normally distributed and expressed as the median (quartile) [M (Q1, Q3)]. Group comparisons were performed using the Mann–Whitney *U* rank-sum test. Univariate logistic regression analysis was conducted to identify potential risk factors for surgical outcomes, and multivariate logistic regression analysis was used to identify independent risk factors. *P* < 0.05 was considered statistically significant.

## Results

### General information before surgery

The study included 110 cases in the recovery group and 9 cases in the restenosis group. Preoperative serum creatinine and blood urea nitrogen levels were measured in mg/dL. A positive result for preoperative urine routine was defined as red blood cells > 30/µL and white blood cells > 40/µL. No significant differences were found in age, gender, BMI, serum creatinine, blood urea nitrogen, and other data between the two groups (*P* > 0.05). However, there were significant differences in preoperative renal pelvis separation, cystatin C, and split renal function (SRF) (*P* < 0.05) (Table 1).

**Table 1:** General information before surgery

Parameter	Recovery group	Restenosis group	${\chi }^{2}$/*t*	*P*
Gender, *n* (%)				
Male	68 (61.82)	8 (88.89)	1.599	0.206
Female	42 (38.18)	1 (11.11)		
Age, *n* (%)				
< 18	11 (10.00)	2 (22.22)	3.988	0.136
18–35	26 (23.64)	4 (44.45)		
> 35	73 (66.36)	3 (33.33)		
BMI $\left( {\overline{x} \pm s} \right)kg/m^2$	24.01 ± 4.22	23.56 ± 3.76	− 0.309	0.758
Affected side, *n* (%)				
Left	60 (54.55)	5 (55.56)	0.000	1.000
Right	50 (45.45)	4 (44.44)		
Preoperative SCR, ($\overline{x}$±$s$) mg/dL	0.84 ± 0.31	0.75 ± 0.28	− 0.839	0.403
Preoperative BUN, ($\overline{x}$±$s$) mg/dL	16.54 ± 4.29	14.87 ± 4.65	− 1.113	0.268
Preoperative BUN/SCR, *n* (%)				
< 20	62 (56.36)	4 (44.44)	0.118	0.732
> 20	48 (43.64)	5 (55.56)		
Previous history of ureteral surgery, *n* (%)				
No	77 (70.00)	6 (66.67)	0.000	1.000
Yes	33 (30.00)	3 (33.33)		
Preoperative renal pelvis resolution, ($\overline{{\varvec{x}}}$±${\varvec{s}}$) mm	38.21 ± 11.89	53.72 ± 18.71	2.448	**0.038**
Preoperative urine routine red blood cells, *n *(%)				
Negative	79 (71.82)	7 (77.78)	0.000	1.000
Positive	31 (28.18)	2 (22.22)		
Preoperative urine routine white blood cells, *n *(%)				
Negative	76 (69.09)	7 (77.78)	0.028	0.867
Positive	34 (30.91)	2 (22.22)		
Cystatin C, mg/L	1.03 ± 0.40	1.66 ± 0.43	4.558	** < 0.001**
SRF, *n *(%)				
< 30%	14 (12.73)	6 (66.67)	13.668	** < 0.001**
> 30%	96 (87.27)	3 (33.33)		

Bold values indicate statistically significant

### Surgical-related information

**Fig. 1:**
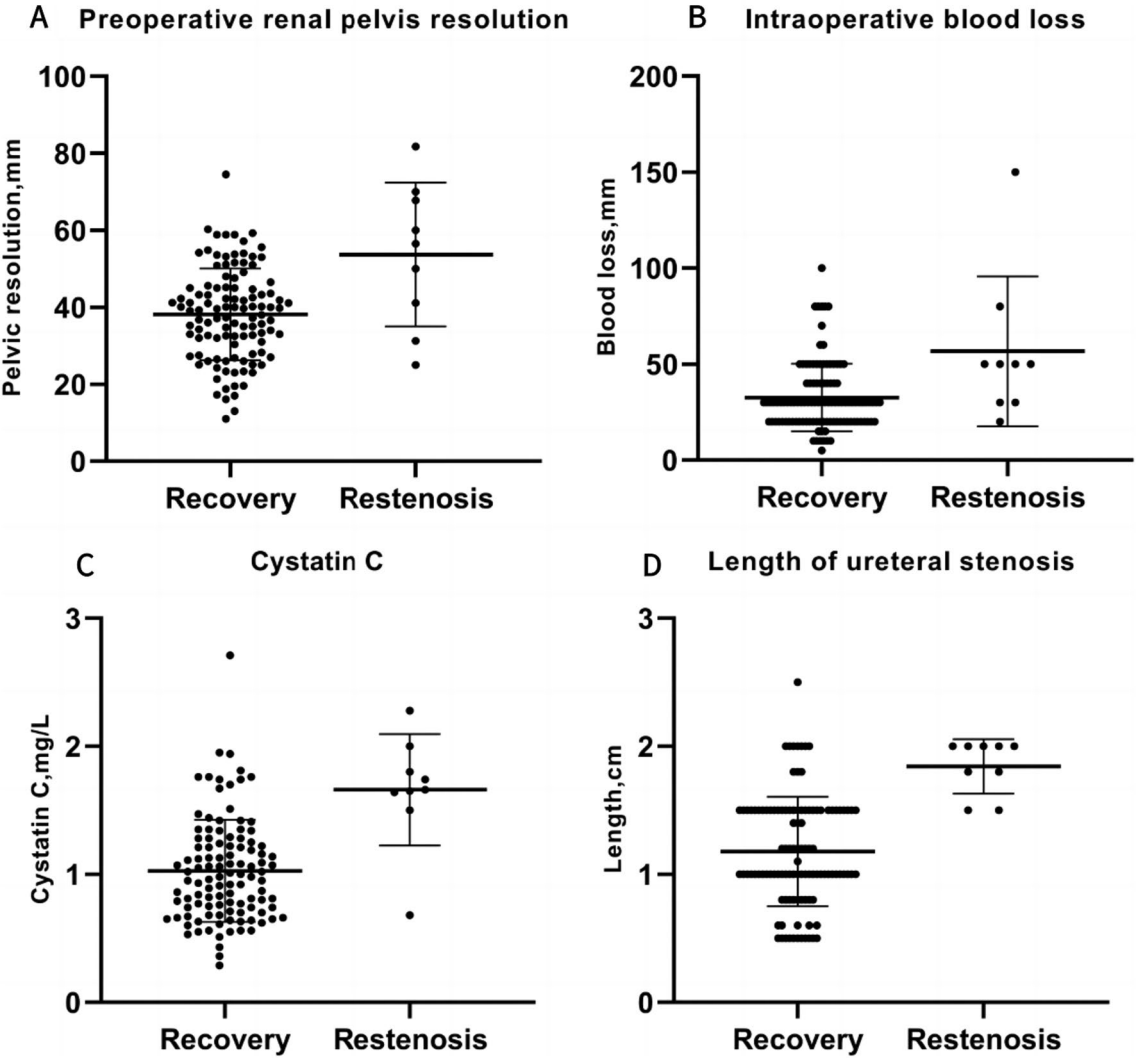
**A** Scatter diagram of separation degree of renal pelvis before operation; **B** scatter diagram of blood loss during operation; **C** scatter diagram of cystatin C before operation; **D** scatter diagram of length of stenosis

When comparing the restenosis group with the recovery group, there were no significant differences in operation time, drainage tube, urinary catheter, double J-tube indwelling time, and perioperative complications (*P* > 0.05). However, the restenosis group had a significantly higher amount of blood loss compared to the improvement group (*P* < 0.05) (Table 2). Figure 1 illustrates the scatter diagrams of preoperative separation of renal pelvis, intraoperative blood loss, preoperative cystatin C, and length of stenosis in the two groups.

**Table 2:** Surgical-related information

Surgical data	Recovery group	Restenosis group	*t/Z*	*P*
Length of ureteral stenosis, ($\overline{x}$±$s$) cm	1.18 ± 0.43	1.84 ± 0.21	8.134	** < 0.001**
Surgery time, ($\overline{x}$±$s$) min	152.85 ± 39.18	179.33 ± 60.40	1.864	0.065
Intraoperative blood loss [M(Q1, Q3), ml]	30.00 (20.00, 40.00)	50.00 (30.00, 65.00)	− 2.508	**0.012**
Drainage tube retention days [M(Q1, Q3), d]	4.00 (3.00, 5.00)	5.00 (3.00, 7.00)	− 0.739	0.460
Urinary catheter retention days [M(Q1, Q3), d]	6.00 (5.00, 8.00)	7.00 (5.50, 8.50)	− 0.820	0.412
Double J-tube retention days [M(Q1, Q3), d]	48.00 (41.00, 57.00)	51.00 (47.50, 54.50)	− 0.920	0.357
Perioperative complications, *n* (%)				
No	88 (80.00)	6 (66.67)	0.269	0.604
Yes	22 (20.00)	3 (33.33)		

Bold values indicate statistically significant

#### Risk factor analysis

**Fig. 2:**
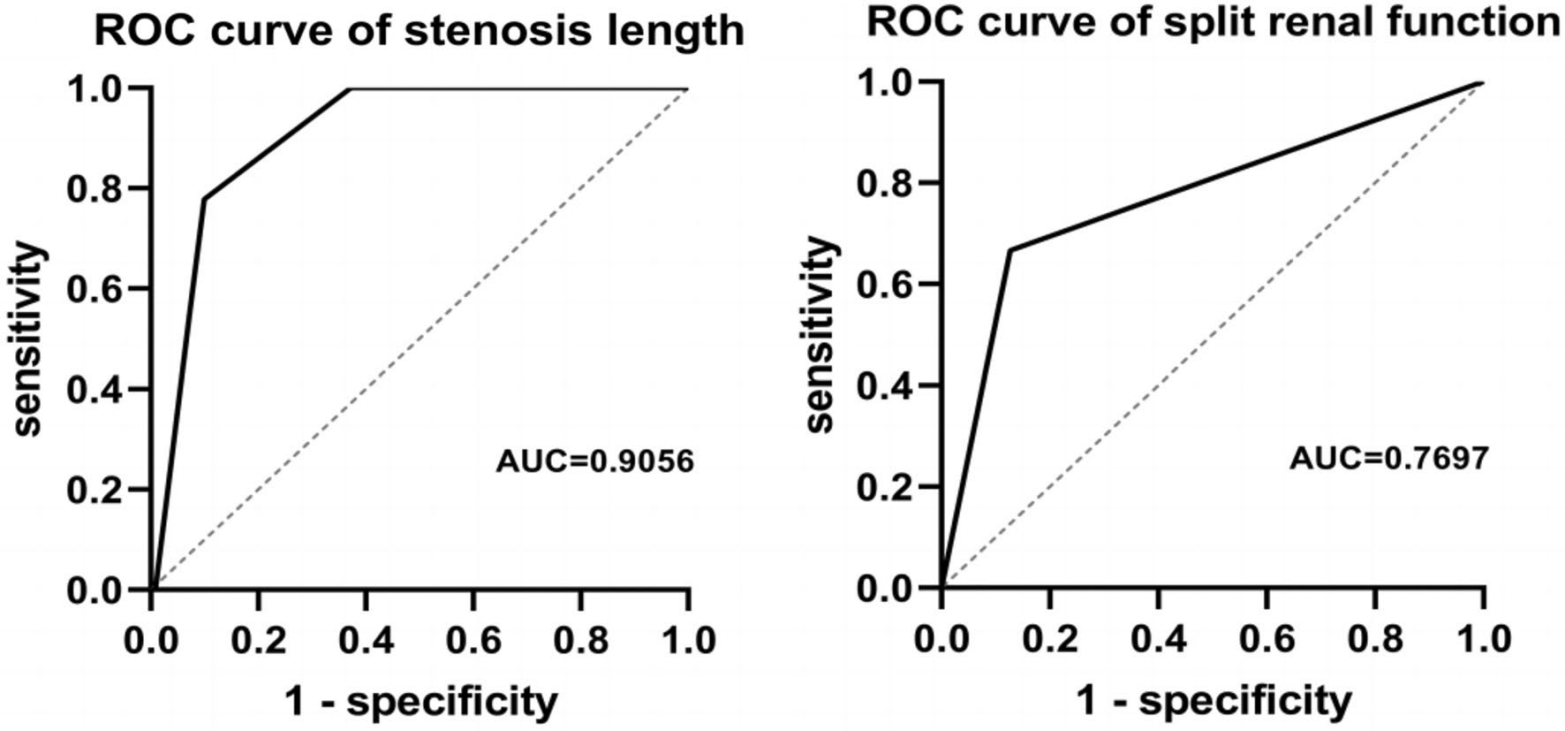
ROC curve of narrow segment length and renal function

All independent variables were diagnosed for collinearity, and no significant collinearity problem was found. Univariate and multivariate logistic regression analyses revealed that the preoperative separation degree of the renal pelvis, cystatin C, and intraoperative blood loss were potential risk factors for postoperative recurrent stenosis. These risk factors were further divided into SRF (odds ratio [OR] = 7.850, *P* = 0.044) and the length of stenosis (OR = 0.025, *P* = 0.011), which was identified as an independent risk factor for postoperative restenosis (Table 3). The ROC curve in Fig. 2 demonstrates that the length of the stenotic segment has a higher predictive ability for postoperative restenosis, with an area under the curve (AUC) of 0.9056, compared to the divided renal function which has an AUC of 0.7697. The cutoff value of the narrow segment length was 1.65 cm. This indicates that the length of the stenotic segment is a better predictor for postoperative restenosis, while the divided SRF can generally predict restenosis.

**Table 3:** Univariate and multivariate logistic analysis of risk factors

Variable	Univariate			Multivariate		
	OR	95% CI	*P* value	OR	95% CI	*P* value
Gender	4.941	0.597–40.925	0.139			
Age						
< 18	–	–	0.171			
18–35	1.182	0.188–7.426	0.859			
> 35	4.424	0.663–29.526	0.125			
BMI	1.027	0.868–1.216	0.755			
Affected side	1.042	0.265–4.088	0.953			
Preoperative SCR	3.946	0.190–82.041	0.375			
Preoperative BUN	1.119	0.919–1.364	0.264			
Preoperative BUN/SCR	0.619	0.158–2.432	0.492			
Previous history of ureteral surgery	0.857	0.202–3.635	0.824			
Preoperative renal pelvis resolution	0.915	0.865–0.969	**0.002**			
Preoperative urine routine red blood cells	1.373	0.270–6.978	0.702			
Preoperative urine routine white blood cells	1.566	0.309–7.933	0.588			
Cystatin C	0.049	0.008–0.285	**0.001**			
SRF	13.714	3.075–61.159	**0.001**	7.850	1.056–58.379	**0.044**
Stenosis length	0.015	0.001–0.164	**0.001**	0.025	0.001–0.424	**0.011**
Surgery time	0.988	0.974–1.001	0.075			
Intraoperative blood loss	0.965	0.939–0.991	**0.008**			
Drainage tube retention days	0.953	0.883–1.027	0.208			
Urinary catheter retention days	0.959	0.890–1.033	0.271			
Double J-tube retention days	0.968	0.928–1.009	0.126			
Perioperative complications	0.500	0.116–2.158	0.353			

Bold values indicate statistically significant

## Discussion

According to the epidemiological survey of patients with UPJO, the incidence of congenital hydronephrosis in children is approximately 1 in 2000. It is more common on the left side than the right side, and the male-to-female ratio is about 2–3 to 1. As one of the most common abnormalities of the urinary system, its impact on children is evident [6, 12]. This study included 13 cases (10.92%) of patients under the age of 18. This could be attributed to the fact that most patients had mild symptoms during their early years and were only incidentally diagnosed during physical examinations or when they presented with obvious symptoms such as waist and abdominal pain. In comparison to children, adults have more developed kidneys, and adult patients with longer duration of obstruction may not have the same postoperative recovery ability as children. Some studies have reported that patients under the age of 35 have faster postoperative recovery compared to older individuals. However, other studies have found no significant effect of age on postoperative outcomes [1, 13, 14]. In our study, patients were divided into three age groups: < 18 years old, 18–35 years old, and > 35 years old. The Chi-square test showed no significant difference in age composition between the two groups (*P* > 0.05). Age composition was not found to be an influencing factor on surgical outcomes (*P* > 0.05). It is important to note that due to the small number of cases in this single-center study, there may be limitations in the conclusions drawn.

Several scholars have reported that patients with low SRF (< 20–40%) may experience poor postoperative recovery after LP. In addition, the level of preoperative SRF can be used to predict the non-restenosis of postoperative renal function [15–18]. Similar to the lack of a standardized indication for surgery in the treatment of UPJO, there is also no unified standard for the cutoff value of SRF that is considered too low. In this study, SRF of 30% was used as the boundary and divided the patients into two groups. Univariate and multivariate logistic regression analysis revealed that SRF < 30% was an independent risk factor for postoperative recurrence of stenosis (OR = 7.850, *P* = 0.044).

The results of regression analysis conducted by Li [13] indicate that the length of the stenosis does not significantly influence the postoperative outcome. However, several studies [10, 19, 20] have reported that an excessively long stenosis (> 2 cm) is likely to result in poor postoperative recovery. Our logistic regression analysis revealed that the length of the stenosis segment is an independent risk factor for postoperative recurrence of stenosis (OR = 0.025, *P* = 0.011). We also determined that the cutoff value for the length of the stenosis segment is 1.65 cm. This implies that if the length of the stenosis segment is greater than 1.65 cm after surgery, the likelihood of relapse will be higher. Furthermore, we observed that the AUC of stenosis length (AUC = 0.9056) is higher compared to renal function (AUC = 0.7697). This suggests that stenosis length has a better predictive ability for postoperative outcomes. This may be attributed to the fact that a longer stenosis requires a larger portion of the ureter to be resected during the operation, resulting in increased tension of the anastomosis and a higher risk of postoperative anastomotic leakage and restenosis.

We observed significant differences between the two groups in preoperative separation of the renal pelvis, cystatin C, SRF, length of stenosis, and intraoperative blood loss (*P* < 0.05). Univariate logistic regression analysis revealed that preoperative separation of the renal pelvis, cystatin C, and intraoperative blood loss are potential risk factors affecting surgical outcomes. The degree of water accumulation may indicate longer obstruction time or severe stenosis, and the increase in cystatin C may be associated with early renal function damage. In addition, greater intraoperative blood loss may suggest more severe ischemia of the ipsilateral ureter, which could result in a poor prognosis. Existing literature [21] supports the notion that excessive hydronephrosis may indicate a poor prognosis. Furthermore, previous studies [1, 18, 22–24] have reported that factors such as obstruction combined with stones, body weight, renal cortex thickness, and ectopic blood vessels may also influence surgical outcomes.

Restenosis after pyeloplasty typically occurs within 1–3 months after the removal of the double J-tube. However, there have been cases of reobstruction reported up to 9–13 years later [25]. Following the removal of the double J-tube, patients are advised to undergo reexamination every 3 months. In this study, four patients did not show significant improvement in hydrops 3 months after the removal of the double J-tube and one patient experienced an increase in the degree of water accumulation after approximately 4 years. It is important to note that the short follow-up period for some patients in this study may result in an underestimation of the actual number of recurrence cases, which may increase over time.

The factors influencing surgery are often complex and multifaceted. This single-center retrospective study has a limited sample size and there are still many influencing factors that have not been considered. While it is important to consider the limitations of the statistical analysis results due to the small sample size, the findings of this study still hold some reference value. This is particularly significant as UPJO patients are relatively rare.

## Conclusion

Logistic regression analysis revealed that the preoperative separation degree of the renal pelvis, cystatin C, and intraoperative blood loss were potential risk factors for postoperative restenosis in LP. In addition, SRF and stenotic segment length were identified as independent risk factors for restenosis. Although recurrent UPJO is uncommon, it poses greater challenges. It is hoped that the research presented in this paper can contribute to clinical decision-making.

## Data Availability

The data that support the findings of this study are available from the corresponding author upon reasonable request.
