# Correction to: Analysis of risk factors for stenosis after laparoscopic pyeloplasty in the treatment of ureteropelvic junction obstruction

**DOI:** 10.1007/s11255-024-04013-9

**Published:** 2024-03-04

**Authors:** Ruilong Chen, Chao Jiang, Xiang Li, Chao Yang, Tengfei Zhu, Yi Wang

**Affiliations:** Department of Urology, The Second Affiliated Hospital of Anhui Medical University, Hefei, 230601 Anhui China

**Correction to: International Urology and Nephrology** 10.1007/s11255-023-03906-5

In this article the affiliation details for all the authors were incorrectly given as ‘Department of Urology, Anhui Medical University Second Hospital, Hefei Economic and Technological Development Zone, No. 678 Furong Road, Hefei 230601, Anhui, China’ but should have been ‘Department of Urology, the Second Affiliated Hospital of Anhui Medical University, Hefei 230601, Anhui, China’.

